# Conducting a medical examination in the COVID-19 era: an Egyptian experience

**DOI:** 10.1186/s43162-021-00038-z

**Published:** 2021-03-01

**Authors:** Ahmed S. Gado, Rawya A. Khater

**Affiliations:** 1 Department of Gastroenterology, Misr International Hospital, Giza, Egypt; 2 Department of Medicine, Cairo University, Giza, Egypt

**Keywords:** COVID- 19, Medical examination, Egyptian Fellowship

## Abstract

**Background:**

On 12 December 2019, a severe respiratory disease was recently reported in Wuhan, known as COVID-19 caused by severe acute respiratory syndrome coronavirus 2. The coronavirus crisis has unequivocally had a marked influence on medical education, particularly in terms of the delivery of assessment. Institutions were forced to implement several changes to medical exam due to COVID-19 pandemic. Written exams were carried out online. Clinical exams were repeatedly canceled or postponed this year. Some institutions carried out clinical exams using remote OSCE stations or without patients. We present our experience in conducting the medical exam in the COVID-19 era.

**Main body:**

Medical exam of the Egyptian Fellowship was canceled in 2020 due to COVID-19 pandemic. The candidates were disappointed and increasingly anxious. After 1 year delay, the internal medicine board decided to carry out the exam after implementing several changes. Changes included the written and clinical exams. The medical exam was successfully conduced in the COVID-19 era.

**Conclusion:**

Conducting a medical exam in the COVID-19 era carries a great challenge for the institutions. Institutions should allow for some degree of flexibility when carrying out exams to prevent suffering of the candidates from the difficult circumstances.

## Background

On 12 December 2019, a severe respiratory disease was recently reported in Wuhan, known as COVID-19 caused by severe acute respiratory syndrome coronavirus 2 [1]. Corona virus is highly contagious and is responsible for the worldwide outbreak pandemic of coronavirus disease [1]. Medical education has experienced unprecedented disruption due to the COVID-19 pandemic. Medical students have had their hospital placements postponed, causing significant anxiety and ushering in a new educational environment which has been difficult to navigate [2]. Exams are notoriously stressful for the candidates. This stress has been compounded by the pandemic’s uncertainty, as candidates have spent their time not knowing if or when their tests would occur [2].

The coronavirus pandemic has had a significant influence on medical education, most notably in terms of the delivery of teaching and assessment. The COVID-19 pandemic triggered new ways of teaching and examinations. Institutions were forced to implement several changes to medical education and exam. Clinical rotations that provide medical students with hospital-based experience have been replaced by online learning and classes have been converted to virtual sessions [2]. The major obstacles have included delivering online teaching content as well as adapting means of assessment in such unforeseen circumstances. In addition to altering teaching provisions, medical schools have also had to adapt their means of assessment. Many universities are switching to online exams during COVD-19. Imperial College London carried out completely remote online written medical exams in which students completed the exam under timed conditions at home [2]. Practical exams or OSCEs are even harder to facilitate remotely and most medical schools were forced to cancel or postpone these until the next academic year. Some institutions carried out clinical exams using remote OSCE stations or without patients. At Imperial, students have now been informed that final year OSCEs next year will include seven remote stations in an attempt to ensure their deliverability [2]. At the Egyptian Fellowship, OSCE stations were carried out without patients in the final clinical exams of surgery and orthopedic. Clinical exam without patients is more likely to be a Viva exam rather than a clinical exam. It assesses knowledge rather than clinical skills. Clinical skills can be judged from the candidate performing the exam in front of the examiner. We present our experience in conducting the medical exam in the COVID-19 era.

## Main text

The internal medicine exam of the Egyptian Fellowship consists of first part and final (exit) exams. The written exam of the first and final exams consists of multiple choice questions, short essay questions, and clinical scenarios. The first part clinical exam consists of four OSCE stations (one history taking and three clinical exams). The final clinical exam consists of one long case exam of 1 h duration and 10 OSCE stations of 10 min duration each (two history taking, two communication skills, and six clinical exams).

Medical exam of the Egyptian Fellowship was canceled in 2020 due to COVID-19 pandemic. The candidates were disappointed and increasingly anxious especially those from foreign countries (Iraq, Yemen, and Sudan) who were planning to return to their countries after the exam. After 1 year delay, the internal medicine board decided to only administer “essential exams.”

Several changes were implemented to the medical exam due to COVID-19 pandemic. The written exams included only multiple choice questions. Short essay questions and clinical scenarios were canceled. First part clinical exam was canceled. In the final clinical exam, long case exam was canceled and the OSCE exam included only eight stations (two history taking, one communication skills, and five clinical exams). History taking and communications skills OSCE stations were carried out without role players in the lecture rooms of the Egyptian Fellowship. Assessors were the role players and the examiners at the same time. Clinical exam was carried out with modified OSCE stations (15 min each) to allow more time for discussion. Clinical exam was carried out in Cairo University Hospital because patients were routinely assessed before admission for COVID-19 disease by non-contrast chest CT scan and specific blood laboratory tests. Most of the admitted patients were acute cases presented mainly with gastrointestinal bleeding and renal failure. Ten OSCE stations were carried out daily for 3 days. One patient refused to continue the exam and was replaced by another patient in the middle of the exam. Two normal persons were used daily due to shortage of suitable cases. Outpatients with chronic diseases were not used in the assessment because of high risk of COVID-19 infection and high cost of doing screening tests. Ten assessors attended the exam daily. All of them were from Cairo University. Fifty-eight candidates were eligible to attend the exam. Some of them were working in COVID-19 quarantine hospitals. According to the university dean instructions, all candidates were screened for COVID-19 infection 72 h before the exam. Oropharyngeal swab was done by RT-PCR assay which was proven to be COVID-19 positive infection in two candidates (3.5%). Both candidates were forbidden to attend the exam. All attendants of the exam were wearing face masks and some were wearing face masks and face shields. Good social distancing practices were strictly applied. Two meters social safe distance was maintained between the assessor and the candidate. No one of the attendants developed any symptoms suggestive of COVID-19 infection.

Fifty-one (91%) candidates passed in the exam. The success rate in the previous year was 58%. The board did not ask for or apply compensation for candidates due to the current circumstances. Feedback was obtained from all assessors about the reasons of the high success rate. Eight (80%) reported sympathy, five (50%) few physical findings in the patients and four (40%) cancelation of long case examination. Feedback was also obtained from the candidates about the changes in the clinical exam. All candidates were happy and satisfied with the cancelation of long case exam.

Conducting a medical exam in the COVID-19 era carries a great challenge for the institutions. With the status of the coronavirus pandemic consistently evolving, the internal medicine board of the Egyptian Fellowship decided that according to the importance of exams the final exam must be held. However, the board acknowledged that there must be a degree of flexibility to conducting the exam, given the unpredictability of the coronavirus and its effect on the future of the candidates. Consequently several changes were implemented to the exam. The medical exam was successfully conduced in the COVID-19 era.

## Conclusion

Conducting a medical exam in the COVID-19 era carries a great challenge for the institutions. Institutions should allow for some degree of flexibility when carrying out exams to prevent suffering of the candidates from the difficult circumstances.

## Data Availability

Not applicable
